# Examining students’ level of understanding toward COVID-19 and its associated factors in Ethiopia: a systematic review and meta-analysis

**DOI:** 10.3389/fpubh.2024.1369738

**Published:** 2024-04-24

**Authors:** Alex Ayenew Chereka, Addisalem Workie Demsash, Fikadu Wake Butta, Adamu Ambachew Shibabaw, Chernet Desalegn Gebeyehu, Daniel Adane, Wubalem Dilie Limeneh, Amare Dagnew Fentahun, Mathias Nega Tadesse, Gemeda Wakgari Kitil

**Affiliations:** ^1^Department of Health Informatics, College of Health Sciences, Mattu University, Mattu, Ethiopia; ^2^Department of Biomedical Science, College of Health Sciences, Mattu University, Mattu, Ethiopia; ^3^Department of Midwifery, College of Medicine and Health Sciences, Injibara University, Injibara, Ethiopia; ^4^Department of Tourism Management, College of Business and Economics, Injibara University, Injibara, Ethiopia; ^5^Department of Computer Science, College of Engineering and Technology, Kebri Dehar University, Kebri Dehar, Ethiopia; ^6^Department of Midwifery, College of Health Sciences, Mattu University, Mattu, Ethiopia

**Keywords:** COVID-19, coronavirus, knowledge, understanding, awareness, students, Ethiopia

## Abstract

**Background:**

This systematic review and meta-analysis aim to investigate students’ understanding of COVID-19 in Ethiopia and identify associated factors. The primary goal is to pinpoint key contributors shaping students’ perception and comprehension of the virus. The study aims to offer valuable insights for developing targeted educational interventions, ultimately enhancing students’ overall knowledge and awareness of the pandemic in the specific context of Ethiopia.

**Methods:**

The study adhered rigorously to PRISMA criteria, ensuring a standardized methodology. Data from reputable databases like Google Scholar and PubMed were systematically collected. Ten relevant articles were meticulously analyzed using STATA version 11, with heterogeneity assessed by the I2 test. A funnel plot and Egger’s test were used to check for publication bias. The determination of the pooled effect size utilized a random-effect model meta-analysis, offering a robust 95% confidence interval.

**Results:**

This meta-analysis, based on 10 articles, reveals an overall prevalence of 61.58% (95% CI: 47.26–75.89). Significant contributors to students’ comprehension include Social media users (AOR) = 2.38, urban residence AOR = 3.31, news media followers AOR = 2.51, fathers’ educational status AOR = 2.35, watching television AOR = 4.71, and health science students AOR = 4.21. These findings underscore crucial elements shaping students’ understanding of COVID-19 in Ethiopia.

**Conclusion:**

Our analysis indicates that 61.58% of Ethiopian students possess a good understanding of COVID-19. Factors such as active social media engagement, geographic location, frequency of news consumption, father’s level of education, television viewing habits, and enrollment in health science programs significantly influence their comprehension. These findings underscore the importance of implementing targeted interventions to enhance health literacy and education among students, thereby facilitating a more effective response to pandemics.

## 1 Background

Coronavirus Disease 2019 (COVID-19) is a highly contagious viral illness caused by the SARS-CoV-2 virus (1). First identified in late 2019 in Wuhan, China, it rapidly spread globally, leading the World Health Organization (WHO) to declare it a pandemic in March 2020 (2, 3). The virus primarily spreads through respiratory droplets, which are released during activities such as coughing, sneezing, or talking. Common symptoms include fever, cough, shortness of breath, and loss of taste or smell (4–6).

The COVID-19 pandemic has touched every aspect of human life worldwide, impacting business, research, education, health, economy, sports, transportation, worship, social interactions, politics, governance, and entertainment (7, 8). This influence affects patients, healthcare workers, communities, and students, spanning all populations (8, 9). The high rate of transmission of COVID-19 makes it challenging to manage its progression (8, 10).

To control the spread of the pandemic globally, various restrictive measures were implemented, such as contact tracing, quarantine, mask-wearing, hand hygiene, stay-at-home orders, lockdowns, and social and physical distancing (10). These measures resulted in the closure of facilities and services, bringing about significant changes to societal norms (11).

As of October 2023, globally, over 698.5 million individuals have been affected by COVID-19, including in Africa and Ethiopia (12). In Africa, the impact extends to more than 12.8 million people (13). In Ethiopia, specifically in October 2023, there were over 501,060 reported cases of COVID-19 (14). The consequences of COVID-19 are profound, affecting healthcare systems, economies, job markets, and education, and causing social and psychological impacts (10). These consequences underscore the contagious and severe nature of the virus (15, 16). This indicates the contagion and severity of the virus for human beings (8).

In March 2020, Ethiopia declared COVID-19 and implemented measures such as social distancing and lockdowns to control the virus’s spread. However, the impact has been profound, straining healthcare systems, disrupting economies, causing job losses, affecting education, and inducing social and psychological impacts (17). Despite the Ethiopian government’s active engagement in testing, contact tracing, treatment, and vaccination campaigns, challenges persist due to limited resources (10, 18).

The global disruption in the education sector caused by the COVID-19 pandemic underscores the importance of understanding how students perceive and interpret the ongoing situation (19). This systematic review and meta-analysis in Ethiopia are specifically designed to delve into students’ knowledge and awareness, while also scrutinizing the factors that shape their perspectives (12). Ethiopia’s unique socio-cultural dynamics contribute a distinctive layer to this investigation, enhancing our understanding of the variations in students’ knowledge levels and consolidating existing knowledge (20).

In light of transformative changes in education, acquiring a nuanced understanding of students’ perspectives is crucial. However, this practice is not widely employed in resource-limited settings (21). Previous studies conducted in Ethiopia have indicated a varying degree of poor knowledge or understanding of COVID-19 among students, with reported outcomes ranging from 23.5 to 86.3% (22–31). This variability could be attributed to factors such as the student’s level of education, attitudes, perceptions, awareness, university or college status, exposure to mass media, place of residence, sources of information, family educational status, and internet and computer accessibility for seeking coronavirus information, among other relevant considerations (21, 22, 25–27, 30, 31).

Despite several studies being conducted on the level of understanding of COVID-19 among Ethiopian students, a systematic review and meta-analysis dedicated specifically to focusing on the students were limited. As a result, this study addresses this limitation, serving as a crucial foundation for informed policymaking, the development of effective public health strategies, and the implementation of targeted educational interventions. The primary objective is to offer valuable insights that contribute to a comprehensive understanding of the impact of the COVID-19 pandemic on Ethiopian students. Ultimately, this study aims to enhance resilience and adaptability among students in the face of persistent challenges.

## 2 Methods

### 2.1 Searching strategy

The systematic review adhered to the Preferred Reporting Items for Systematic Reviews and Meta-Analysis (PRISMA) guidelines (32). Between October 1 and November 10, 2023, we conducted a thorough data search across various platforms, including PubMed, the Cochrane Library, Google, Google Scholar, and the Embase database. This search encompassed both Medical Subject Headings (MeSH) and free-text terms, employing a detailed search strategy.

To enhance the search precision, the systematic review employed both “AND” and “OR” in the search strategy. In addition to the mainstream sources, the Master’s thesis or Dissertation was explored through the Ethiopian university’s research repository online library. Furthermore, a manual search of the reference lists in the included studies was conducted to ensure a comprehensive coverage of evidence.

To comprehensively examine the level of understanding among students regarding COVID-19 and the influencing factors in Ethiopia, we employed a thorough search strategy. Utilizing snowballing techniques, we explored various search phrases, including keywords and free-text queries.

We utilized a systematic approach to search relevant literature across multiple electronic databases including PubMed, Web of Science, Scopus, and Google Scholar. The search terms were carefully selected to capture all relevant studies related to students’ understanding of COVID-19 and its associated factors in Ethiopia.

Specifically, our search strategy included keywords such as “COVID-19,” “students,” “knowledge,” “awareness,” “Ethiopia,” and variations thereof. Boolean operators (e.g., AND, OR) were utilized to combine these keywords effectively. Additionally, Medical Subject Headings (MeSH) terms were employed where applicable to ensure a comprehensive search strategy.

### 2.2 Inclusion and exclusion criteria

The inclusion criteria for this systematic review encompassed freely accessible full-text articles conducted in Ethiopia between 2019 and 2023 and written in English. We included studies that underwent peer review for publication in journals or were discovered in gray literature. Eligible studies covered all observational study designs (cross-sectional, case–control, and cohort) involving human subjects and reporting on knowledge, understanding, and awareness of COVID-19 in their full articles. To ensure inclusivity, we adopted a comprehensive approach, welcoming a diverse range of observational study designs and sources. These criteria were established *a priori* to ensure the selection of studies that met the objectives of our systematic review. Studies were included if they met the following criteria: (1) conducted in Ethiopia, (2) focused on assessing students’ understanding or knowledge of COVID-19, (3) published in peer-reviewed journals, and (4) written in English.

However, studies without freely accessible full texts after applying the Preferred Reporting Items for Systematic Reviews and Meta-Analysis Protocols search strategies were excluded. Furthermore, studies not quantitatively reporting specific proportions of good knowledge, understanding, and awareness regarding COVID-19 were also excluded from this systematic review and meta-analysis. This stringent approach aimed to concentrate on studies offering detailed and quantitative insights into the specified aspects of COVID-19 awareness and understanding in the Ethiopian context. Studies were excluded if they were duplicates, conference abstracts, editorials, or opinion pieces.

### 2.3 Outcome measures

The primary goal of this systematic review and meta-analysis was to estimate the combined level of students’ knowledge, understanding, or awareness regarding COVID-19 in Ethiopia. We categorized students’ knowledge into either good or poor responses during the assessment process.

### 2.4 Data extraction

Following the selection criteria, two authors independently reviewed titles and abstracts. Selected titles and abstracts underwent further scrutiny, and data extraction was carried out using an organized Microsoft Excel Spreadsheet. Studies approved by both authors during this selection process were included in the review. In case of disagreements among data extractors, discussions were held to reach a consensus.

For each study, comprehensive information was collected, including the initial author, publication year, study years, participant count, background details, study area, sample size, data collection techniques, response rate, study design, and prevalence. The status of Ethiopian students’ knowledge and associated factors was also extracted, along with 95% confidence intervals. Any identified issues were discussed with the corresponding author for resolution.

### 2.5 Quality appraisal of selected literature

The quality appraisal of the selected literature involved a thorough assessment to ensure the reliability and validity of the studies included in the systematic review. The evaluation process considered various criteria such as study design, methodology, sample size, data collection techniques, and the overall rigor of each study.

Using a standardized tool (a modified version of the Newcastle-Ottawa Scale (NOS)), that categorizes bias potential and can help to explain variations in the results of included research, Each study’s quality was assessed, and the authors additionally examined at each publication’s methodology and other features (33). We concluded that works with a modified NOS component score of 7 or higher were relevant after analyzing a range of publications (see: Table 1) (34). Additionally, three authors independently conducted a quality control assessment.

**Table 1:** Characteristics of individual studies conducted on the level of understanding towards COVID-19 among students in Ethiopia, 2024.

**Authors**	**Regions**	**Year of the study**	**Year of publication**	**Study design**	**Sample size**	**Prevalence**	**Quality score**
Aklil, M. and Temesgan, W.	Amhara	2021	2022	Cross-sectional	634	46.8	9
Aynalem, Y. et al.	Amhara	2020	2021	Cross-sectional	634	73.8	8
Feleke, A. et al.	Amhara	2021	2022	Cross-sectional	417	98.8	9
Getawa, S. et al.	Amhara	2021	2022	Cross-sectional	395	93.6	8
Yesuf, M. and Abdu, M.	SNNP	2021	2022	Cross-sectional	422	81.8	7
Berihun, G. et al.	Amhara	2020	2021	Cross-sectional	422	75.9	9
Handebo, S. et al.	Amhara	2020	2021	Cross-sectional	403	23.5	7
Larebo, M. and Abame, E.	SNNP	2021	2021	Cross-sectional	800	29.2	8
Angelo, T. et al.	SNNP	2020	2021	Cross-sectional	422	47	8
Tadesse, W. et al.	Amhara	2020	2020	Cross-sectional	422	69.6	8

### 2.6 Data processing and analysis

The data processing and analysis employed a systematic approach to extract meaningful insights. Initially, data was meticulously organized in Microsoft Excel and then imported into STATA version 11, for further analysis. In the analytical phase, a random-effects model of meta-analysis estimated both the pooled and individual study effects, accompanied by 95% confidence intervals (CI) for a nuanced understanding. The visual representation used forest plots, providing a clear overview of the pooled impact size and weight, complemented by 95% CI for each selected study.

Ensuring result validity involved assessing heterogeneity among studies using I2 statistics (35), considering variations in participants, locations, and measurement methods. A random-effects model was chosen for flexibility in handling identified heterogeneity. A meticulous check for publication bias was conducted using funnel plots and Egger’s test to enhance credibility by addressing potential bias in the published literature (34). In essence, this methodology aimed to deliver a comprehensive, reliable, and unbiased evaluation of Ethiopian students’ knowledge levels concerning COVID-19.

## 3 Patient and public involvement

No patients were involved in developing the research question, determining outcome measures, designing the study, recruiting participants, analyzing data, interpreting results, or implementing the research. Furthermore, the study design did not directly engage the general public, and there is no intention to share the findings with patients.

### 3.1 Ethical consideration

As part of this systematic review, we applied the Preferred Reporting Items for Systematic Reviews and Meta-Analysis (PRISMA) criteria to evaluate the literature. To address potential conflicts of interest, as well as voice and representation issues, we systematically reviewed and acknowledged the included research in the manuscript. Therefore, the dates of participant recruitment and/or medical record access are not relevant, and ethics approval is not applicable.

## 4 Results

### 4.1 The selection process of the articles

In the process of conducting a comprehensive literature review, various online search engines, including Google, Google Scholar, and other databases such as Medline, Pub Med, Scopus, Cochrane, EMBASE, African Journal Online (AJOL), HINARI, and Science Direct, were utilized. This extensive search yielded a total of 18,300 papers across all databases. To ensure quality and relevance, a meticulous screening process unfolded. Initially, 4,500 duplicate papers were removed. Subsequently, 8,327 papers were disqualified based on a detailed review of abstracts and titles, considering factors like study focus and subject matter.

**Figure 1:**
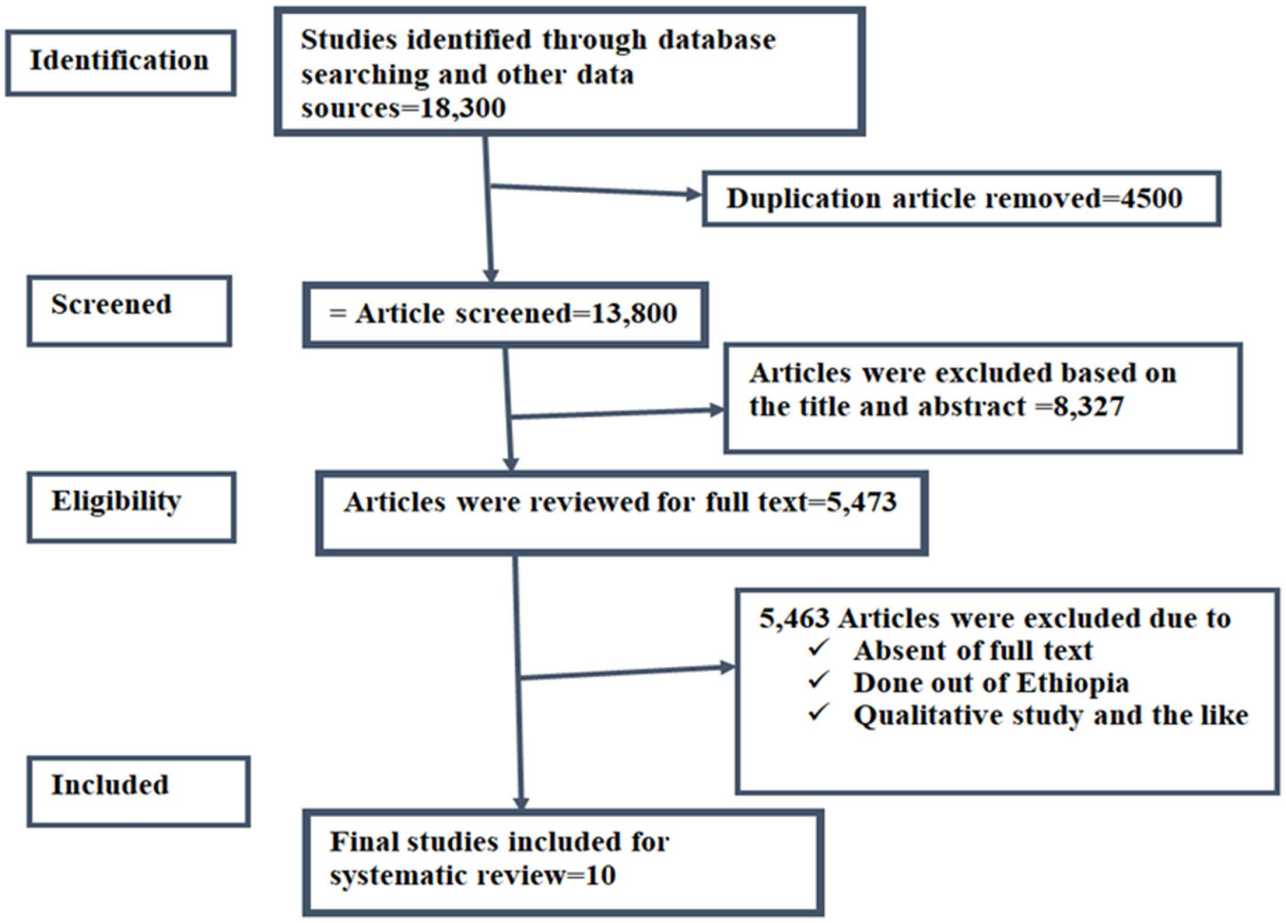
PRISMA flowcharts showing the selection process of the articles.

Refinement continued with the exclusion of 183 publications due to quality and full-text availability issues. Subsequently, 5,280 full-text papers were excluded based on study area and subject matter, refining the focus. The research specifically centered on Ethiopia, involving a meticulous meta-analysis and systematic review of 10 carefully chosen full-text papers. This thorough process ensures a robust and meaningful analysis, providing valuable insights into the study’s specific context (see Figure 1).

### 4.2 Characteristics of the included articles in the review

This study, encompassing 10 articles and involving 5,003 study participants, aimed to assess the collective understanding of students regarding COVID-19 in Ethiopian educational sectors. The majority of the studies, analyzed through a systematic review and meta-analysis, were conducted in the Amhara region, with study participants varying from 403 to 634 (25–27, 29–31). Additionally, three articles focused on the South nation nationality people, involving study participants ranging from 422 to 800. The inclusion of diverse regions provided a comprehensive perspective on students’ comprehension of COVID-19 in the Ethiopian educational context (23, 24, 28).

The variability in students’ understanding of COVID-19 in Ethiopia was substantial, ranging from 23.5 to 86.3% across the included articles. Importantly, all reviewed studies met a specified quality threshold of seven and above on a modified version of the Newcastle-Ottawa Scale (NOS). This comprehensive analysis provides valuable insights into the diverse perspectives and comprehension levels among students in different regions of Ethiopia, offering crucial information for the formulation of effective educational and public health strategies (see Table 1).

### 4.3 The pooled prevalence of Ethiopian students’ level of understanding of COVID-19

According to the reviewed articles, the level of understanding among students in the Ethiopian educational sector regarding COVID-19 was found to be insufficient. The meta-data analysis revealed that the pooled prevalence of students with a good level of understanding of COVID-19 in Ethiopia was 61.58% (95% CI, 47.26–75.89). However, a random-effects model showed a statistically significant level of heterogeneity (I2 = 70.8%; *p* = 0.000), indicating substantial variation among the primary studies.

**Figure 2:**
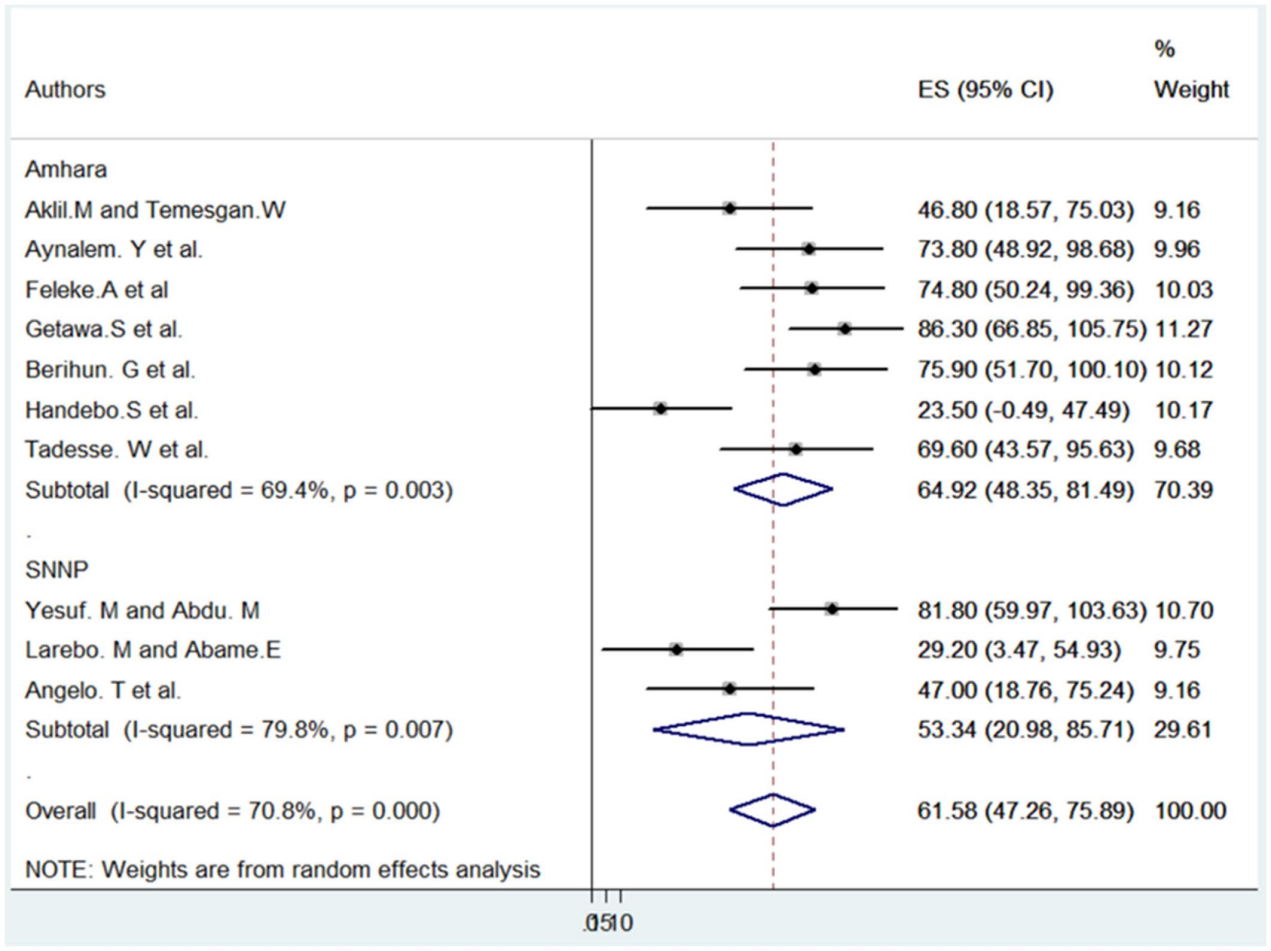
The pooled prevalence of good levels of understanding towards COVID-19 among students in Ethiopia, 2024.

Consequently, the conclusion drawn from this significant heterogeneity was that further investigation through subgroup analysis is warranted. This suggests that there may be underlying factors contributing to the diversity in students’ understanding of COVID-19 in Ethiopia, and exploring specific subgroups or factors could provide valuable insights for addressing these variations (see Figure 2).

### 4.4 Publication bias

In this meta-analysis, both Egger’s regression test and funnel plots were employed to assess the presence of publication bias. Unfortunately, the results from the funnel plots indicated evidence of publication bias, as the observed asymmetry contradicted the expected symmetrical distribution. It’s crucial to note that each point in the funnel plot represents a visual representation of study results rather than individual studies. Despite the anticipation of symmetry, the observed asymmetry raised concerns about potential biases in the included studies.

**Figure 3:**
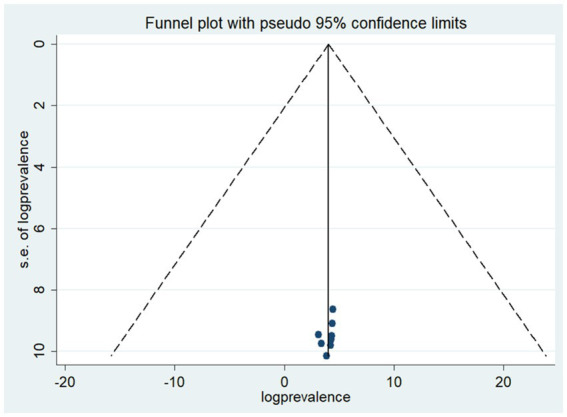
Funnel plot to shows publication bias among the included articles of a good level of understanding towards COVID-19 a systematic review and meta-analysis, Ethiopia.

Furthermore, Egger’s regression test, evaluating the statistical significance of publication bias, yielded a result of *p* = 0.042. This *p*-value, being less than the conventional significance level (e.g., 0.05), indicates that Egger’s test for the absence of publication bias was not statistically significant. In summary, both the visual inspection of the funnel plot and the statistical test collectively suggest the presence of publication bias in this meta-analysis (see Figure 3).

### 4.5 Univariate meta-regression of factors related to the heterogeneity of students’ level of understanding toward COVID-19 in Ethiopia

In our study, we initially conducted a univariate meta-analysis, focusing on response rate and the year of publication, to investigate potential causes of heterogeneity among the collected data. Surprisingly, this analysis did not reveal a significant link between either response rate or publication year and the observed heterogeneity.

To delve deeper into understanding the sources of variation, we proceeded with a subgroup analysis. This involved considering additional factors such as the study’s area, sample size, years of publication, and the specific year of the study. Despite this thorough exploration, none of these factors emerged as a potential cause of the observed heterogeneity.

In essence, our thorough examination did not pinpoint response rate or year of publication, nor did it reveal any other specific factors considered in the subgroup analysis, as significant contributors to the heterogeneity observed in the studies. This suggests that further exploration or the consideration of other factors may be necessary to understand the sources of variability in the meta-analysis (see Table 2).

**Table 2:** Univariate Meta-regression of factors related to heterogeneity level of understanding towards COVID-19, systematic review and meta-analysis, Ethiopia, 2024.

**Factors**	**Coefficient**	***p*-value**
Response rate	0.1372339	0.874
Year of publication	10.553313	0.427

### 4.6 Subgroup analysis

In our systematic review and meta-analysis, we encountered significant variability among the articles included. To better understand and address this heterogeneity, we conducted a subgroup analysis, considering key factors such as the study region, sample size, study area, year of publication, and year of the study.

Upon analyzing these subgroups, we found that the primary source of heterogeneity was studies conducted in secondary schools (*I*^2^ = 83.7%, *p* = 0.003). The second most significant source of heterogeneity was attributed to studies conducted in the Amhara region (*I*^2^ = 79.8%, *p* = 0.007), while the third major source was studies conducted after the year 2021 (*I*^2^ = 75.2%, p = 0.003).

**Figure 4:**
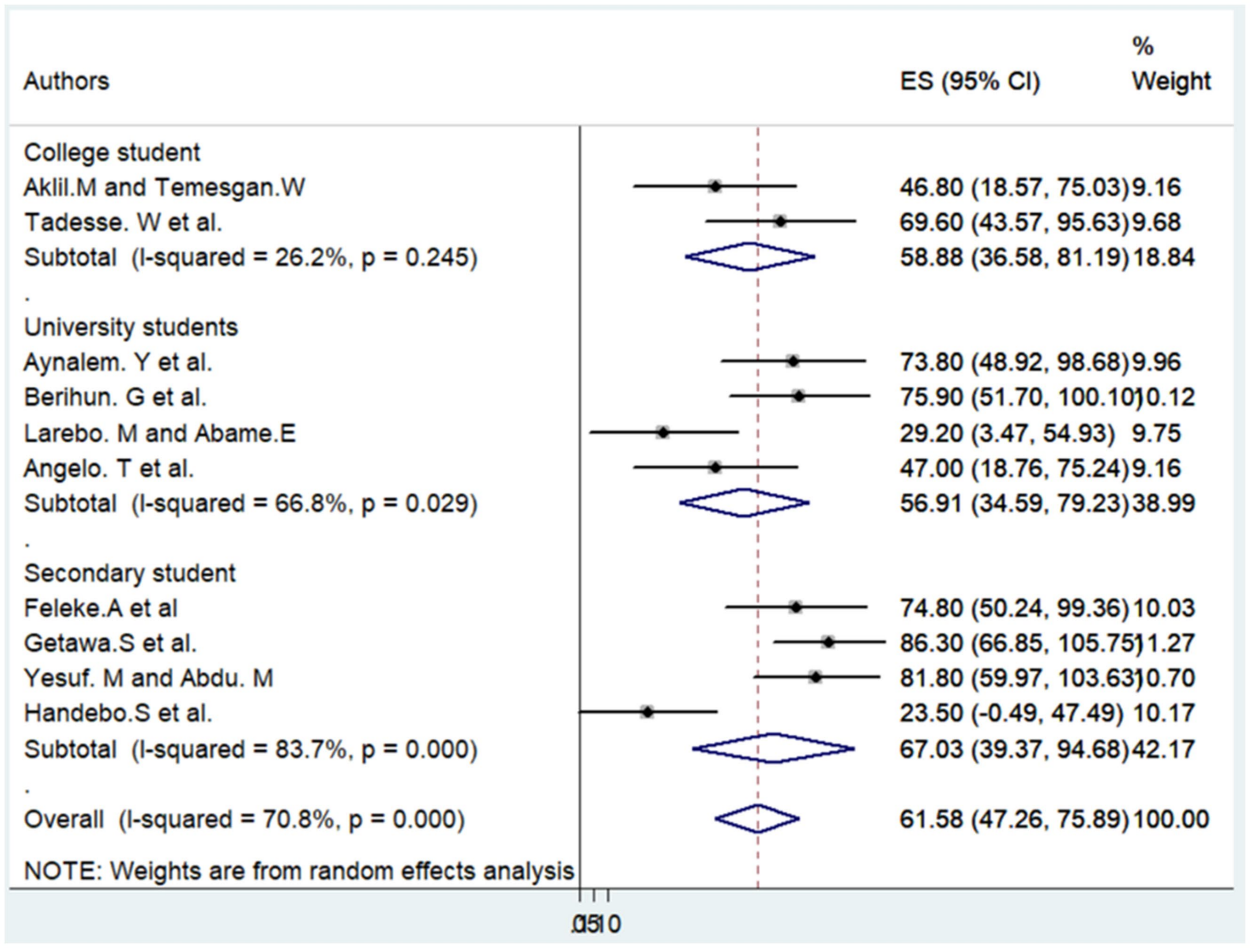
Subgroup analysis based on study area.

It’s noteworthy that all other variables considered in the subgroup analysis also contributed to the overall heterogeneity. This comprehensive examination provides valuable insights into the specific factors influencing the variability observed in our meta-analysis, with a clear hierarchy of influence identified (see: Table 3 and Figure 4).

**Table 3:** Sub-group meta-analysis of factors relating to the heterogeneity of students’ level of understanding towards COVID-19.

**Variables**	**Category**	**Included study**	**Sample size**	**Prevalence**	***I*** ^**2**^	***p*-value**
Study area	University	4	2,278	56.92	66.8%	0.029
	College	2	1,056	58.88	26.2%	0.245
	Secondary school	4	1,669	67.03	83.7%	0.000
Sample size	Large sample size	3	2068	50.17	67.0%	0.048
	Small sample size	7	2,935	66.58	71.4%	0.002
Year of study	After 2021	5	2,700	64.91	75.2%	0.003
	Before 2021	5	2,303	57.98	69.1%	0.000

### 4.7 Factors associated with Ethiopian student’s level of understanding of COVID-19

This study, based on an analysis of 10 articles, aimed to uncover the factors influencing students’ understanding of COVID-19 in Ethiopia. Six key factors were identified. Firstly, students active on social media showed a higher understanding (AOR = 2.38). Additionally, residents of specific locations demonstrated significantly better comprehension (AOR = 3.31). Those who followed news media also exhibited a stronger grasp of COVID-19-related information (AOR = 2.51). The educational status of fathers was linked to students’ understanding (AOR = 2.35), and those who engaged in television watching showed notably higher comprehension (AOR = 4.71). Lastly, health science students demonstrated a greater understanding (AOR = 4.21). In conclusion, the study underscores the diverse factors influencing students’ COVID-19 understanding in Ethiopia, spanning media engagement, family background, academic discipline, and personal circumstances.

**Figure 5:**
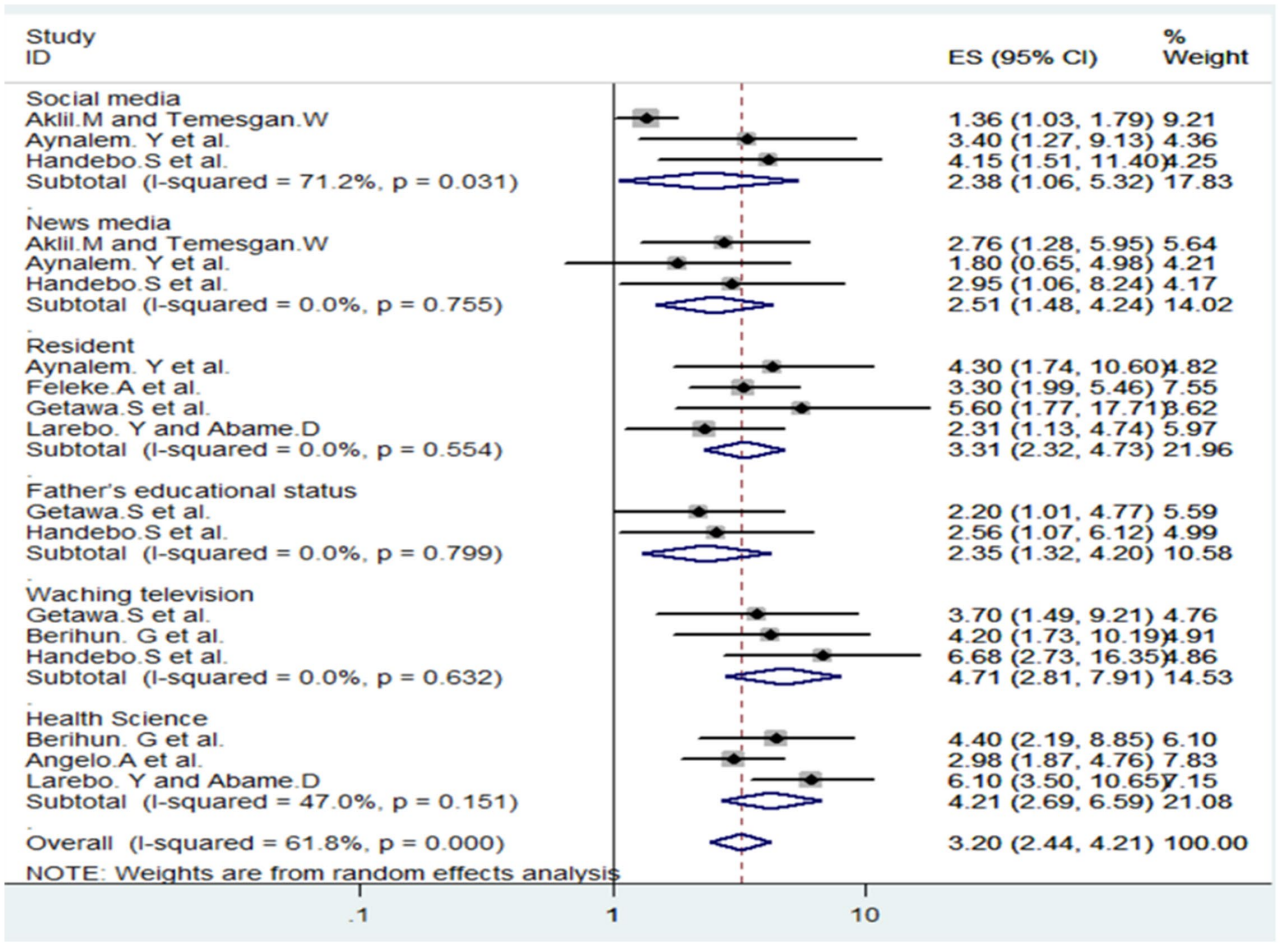
Factors Associated with Ethiopian Student’s Level of Understanding towards COVID-19.

In total, four studies were accessed to assess the correlation between students’ level of understanding regarding COVID-19 and their residence. Another set of three studies investigated the relationship between students’ comprehension of COVID-19 and factors such as being social media users, followers of news media, and respondents who watch television. Additionally, these studies explored the understanding of students majoring in health science departments in college and university study areas. Finally, two studies delved into the impact of marital status and fathers’ educational status on students’ level of understanding of COVID-19. Collectively, these studies provide a comprehensive analysis of various factors influencing students’ understanding of the pandemic, contributing valuable insights across different dimensions of their lives and academic pursuits (see Figure 5).

## 5 Discussion

This systematic review and meta-analysis of students’ understanding of COVID-19 in Ethiopia revealed a pooled prevalence of 61.58% (95% CI: 47.26–75.89). This indicates that a substantial majority of students possess a reasonable level of comprehension. The study emphasizes the importance of targeted interventions considering diverse factors influencing understanding, providing valuable insights for educational strategies in the context of the ongoing pandemic.

Our study aligns with previous research conducted in Ethiopia among pregnant women, reporting a prevalence of 60.24% (36), and in the overall population, including healthcare professionals and students, with a prevalence of 61.78% (37). However, it notes a lower prevalence compared to similar studies conducted among healthcare professionals in Ethiopia, where the prevalence was 79.4% (38), and in the general population, including nearly 75% of healthcare professionals, with a prevalence of 70.25% (39).

This variation may be explained by the fact that healthcare professionals benefit from their educational backgrounds and continuous exposure to professional training, specialized resources, and ongoing updates within the healthcare environment. This heightened access contributes to a more comprehensive understanding of COVID-19 among this group.

Based on the associated factors analysis, six significant factors influence students’ understanding of COVID-19 in Ethiopia. Among these, regular television watching emerged as the most significant factor. Students who engage in this activity are 4.71 times more likely to grasp information compared to those who do not watch regularly. Television, serving as a common source of information, aids students by providing accessible and comprehensive details about the virus. This was supported by studies done (22, 25, 30). One possible justification is that students who utilize television as a source of information regarding COVID-19 play a vital role in expanding their understanding of the pandemic. Television’s widespread accessibility ensures that a diverse range of students, including those from various socioeconomic backgrounds, can access timely and relevant information. Moreover, the visual and auditory presentation offered by television facilitates comprehension and retention of complex topics like COVID-19 through news broadcasts, documentaries, and expert interviews. Visual aids such as charts and graphs further enhance understanding. Additionally, many students trust established television networks for accurate information, as these networks often feature dedicated health segments and special coverage on COVID-19, including updates from reputable sources like the World Health Organization and the Centers for Disease Control and Prevention. The consistency and routine of regular television watching also contribute to students’ knowledge acquisition, as it establishes a habit of seeking out information on the pandemic. Research demonstrates that the frequency of television watching correlates with the depth of understanding; students who watch television regularly are 4.71 times more likely to grasp information about COVID-19 compared to those who do not. In conclusion, students who rely on television for COVID-19 updates significantly enhance their knowledge base, benefiting from television’s accessibility, presentation style, trusted networks, consistency, and frequency of exposure.

The second most significant factor influencing students’ understanding of the pandemic is enrollment in health science departments. Students in these departments are 4.21 times more likely to have a deep understanding compared to those outside health science departments. This is attributed to the focus on health-related subjects within these departments, providing students with more in-depth knowledge about COVID-19. This is in line with studies done (25).

Living in urban areas also emerged as a significant factor, with students in cities being 3.31 times more likely to possess a thorough understanding compared to students living in rural areas, this could be attributed to urban settings offering increased access to information, and healthcare facilities, and generally providing a higher educational environment. This is in line with the study done (36).

Active engagement with news media significantly influences students’ understanding of the pandemic, with a 2.51 times higher likelihood for those who actively follow news media to have a solid understanding. This highlights the importance of regularly exposing oneself to trustworthy news sources, as it plays a crucial role in making students more informed about COVID-19. Regular and reliable information from news media contributes significantly to students’ knowledge and comprehension of the ongoing pandemic (38).

Active engagement in social media plays a significant role in shaping students’ understanding of COVID-19. Those who actively participate in social media are 2.38 times more likely to comprehend the situation compared to those who do not engage in social media. The swift dissemination of information through social media offers diverse perspectives and real-time updates, contributing to a more comprehensive awareness among students (38).

Lastly, the educational background of fathers emerged as a significant factor, suggesting that students with fathers having higher education are 2.35 times more likely to understand COVID-19 comprehensively. This highlights the important role of family educational environments in shaping students’ knowledge, emphasizing the interplay of familial factors in educational outcomes. In summary, these factors collectively play a crucial role in shaping how well students understand COVID-19 in Ethiopia. This is in line with the study done (36).

### 5.1 Strengths and limitations of the study

The strength of our study lies in its comprehensive examination of students’ understanding of COVID-19 in Ethiopia through a systematic review and meta-analysis. By synthesizing data from various sources, we obtained a broad perspective on this crucial subject, contributing to a nuanced understanding of the factors influencing students’ comprehension. Our meticulous approach to screening, excluding duplicates, and assessing study quality enhances the reliability of our findings, ensuring a robust foundation for analysis.

However, it’s important to acknowledge certain limitations. The primary weakness arises from the inherent constraints of the available literature. Variability in study designs, data reporting, and regional differences posed challenges in standardizing the analysis. Additionally, the exclusion of studies in languages other than English may introduce a language bias. Moreover, the evolving nature of the pandemic may render some data outdated, affecting the timeliness of our conclusions. Despite these limitations, our study provides valuable insights into students’ understanding of COVID-19 in Ethiopia, offering a foundation for targeted educational interventions and avenues for further research.

## 6 Conclusion and recommendations

This systematic review and meta-analysis on students’ understanding of COVID-19 in Ethiopia identified several significant factors, including social media usage, urban residence, news media followership, fathers’ educational status, television watching, and enrollment in health science programs. This comprehensive understanding underscores the complex interplay of various factors shaping students’ knowledge of COVID-19 in Ethiopia.

In light of the significant factors identified in this study, policymakers, educators, and public health authorities in Ethiopia must adopt a multifaceted approach to enhance students’ knowledge and awareness of COVID-19. Given the complexity of factors influencing students’ understanding, interventions should be tailored to address the diverse socio-demographic characteristics and media consumption patterns observed among students.

### 6.1 Targeted educational campaigns

Develop targeted educational campaigns aimed at increasing awareness and knowledge of COVID-19 among students, particularly those residing in urban areas and enrolled in health science programs. These campaigns should leverage various communication channels, including social media, television, and news media, to disseminate accurate and up-to-date information about COVID-19 prevention, transmission, and mitigation strategies.

### 6.2 Engagement with social media platforms

Collaborate with social media platforms to promote reliable sources of information and combat misinformation surrounding COVID-19. Encourage students to critically evaluate the information they encounter on social media and provide resources for fact-checking and verifying the accuracy of COVID-19-related content.

### 6.3 Incorporation into curriculum

Integrate COVID-19 education into the curriculum of health science programs and other relevant disciplines. Incorporate evidence-based teaching materials and case studies that highlight the socio-economic determinants of COVID-19 knowledge and its implications for public health practice in Ethiopia.

### 6.4 Community engagement

Foster community engagement initiatives that involve students, parents, teachers, and local leaders in discussions about COVID-19 awareness and prevention. Encourage open dialog and collaboration to address misconceptions, cultural beliefs, and barriers to accessing accurate information about the pandemic.

### 6.5 Capacity building

Invest in capacity-building initiatives to empower students with the skills and knowledge needed to become advocates for COVID-19 prevention and health promotion within their communities. Provide training opportunities on effective communication strategies, health literacy, and community outreach techniques.

## Data availability statement

The original contributions presented in the study are included in the article/Supplementary material, further inquiries can be directed to the corresponding author.

## Author contributions

AC: Conceptualization, Data curation, Formal analysis, Funding acquisition, Investigation, Methodology, Project administration. AD: Conceptualization, Data curation, Supervision, Validation, Writing – original draft, Writing – review & editing. FB: Conceptualization, Data curation, Formal analysis, Investigation, Validation, Visualization, Writing – original draft, Writing – review & editing. AS: Formal analysis, Methodology, Validation, Visualization, Writing – original draft, Writing – review & editing. CG: Conceptualization, Data curation, Investigation, Methodology, Validation, Visualization, Writing – original draft, Writing – review & editing. DA: Conceptualization, Validation, Visualization, Writing – original draft, Writing – review & editing. WL: Data curation, Formal analysis, Validation, Visualization, Writing – original draft, Writing – review & editing. AF: Conceptualization, Investigation, Software, Validation, Visualization, Writing – original draft, Writing – review & editing. MT: Conceptualization, Software, Supervision, Validation, Visualization, Writing – original draft, Writing – review & editing. GK: Conceptualization, Investigation, Methodology, Data curation, Formal analysis, Supervision, Validation, Visualization, Writing – original draft, Writing – review & editing.

## References

[1] Peng Y Pei C Zheng Y Wang J Zhang K Zheng Z . A cross-sectional survey of knowledge, attitude, and practice associated with COVID-19 among undergraduate students in China. BMC Public Health. (2020) 20:1–8. doi: 10.1186/s12889-020-09392-z 32847554 PMC7447607

[2] Zhu N Zhang D Wang W Li X Yang B Song J . A novel coronavirus from patients with pneumonia in China, 2019. N Engl J Med. (2020) 382:727–33. doi: 10.1056/NEJMoa2001017, PMID: 31978945 PMC7092803

[3] World Health Organization and World Health Organization, nCoV outbreak is an emergency of international concern. (2019).

[4] Wang D Hu B Hu C Zhu F Liu X Zhang J . Clinical characteristics of 138 hospitalized patients with 2019 novel coronavirus–infected pneumonia in Wuhan, China. JAMA. (2020) 323:1061–9. doi: 10.1001/jama.2020.1585, PMID: 32031570 PMC7042881

[5] Chen N Zhou M Dong X Qu J Gong F Han Y . Epidemiological and clinical characteristics of 99 cases of 2019 novel coronavirus pneumonia in Wuhan, China: a descriptive study. Lancet. (2020) 395:507–13. doi: 10.1016/S0140-6736(20)30211-7, PMID: 32007143 PMC7135076

[6] WHO. COVID-19 weekly epidemiological update, 3 November 2020. (2020).

[7] Anteneh RM Dessie AM Azanaw MM Anley DT Melese BD Feleke SF . The psychological impact of COVID-19 pandemic and associated factors among college and university students in Ethiopia: a systematic review and meta-analysis, 2022. Front Public Health. (2023) 11:1136031. doi: 10.3389/fpubh.2023.1136031, PMID: 37521996 PMC10374415

[8] Chereka AA Gashu KD Fentahun A Tilahun B Fikadie B Ngusie HS. COVID-19 related knowledge sharing practice and associated factors among healthcare providers worked in COVID-19 treatment centers at teaching hospitals in Northwest Ethiopia: a cross-sectional study. Inform Med Unlock. (2022) 28:100856. doi: 10.1016/j.imu.2022.100856, PMID: 35071731 PMC8760095

[9] Browning MH Larson LR Sharaievska I Rigolon A McAnirlin O Mullenbach L . Psychological impacts from COVID-19 among university students: risk factors across seven states in the United States. PLoS One. (2021) 16:e0245327. doi: 10.1371/journal.pone.0245327, PMID: 33411812 PMC7790395

[10] Chereka A.A. Demsash A.W. Ngusie H.S. Kassie S.Y., Digital health literacy to share COVID-19 related information and associated factors among healthcare providers worked at COVID-19 treatment centers in Amhara region, Ethiopia: A cross-sectional survey. Informatics in Medicine Unlocked. (2022). 30:100934. 35441087 10.1016/j.imu.2022.100934 PMC9010014

[11] WHO. COVID-19 weekly epidemiological update, 22 December 2020. (2020).

[12] WHO. Available at: https://www.worldometers.info/coronavirus/?utm_campaign=homeAdvegas1?#countries. (2019).

[13] WHO. *Coronavirus disease (COVID-2019) situation reports*. (2020).

[14] WHO. *COVID-19 weekly epidemiological update, edition 58, 21 September 2021.* (2021).

[15] Ph AMAR . SARS-CoV-2 outbreak: how can pharmacists help? Res Soc Adm Pharm. (2021) 17:480–2. 10.1016/j.sapharm.2020.03.018 PMC7271257 32241695

[16] Khan Z Muhammad K Ahmed A Rahman H. Coronavirus outbreaks: prevention and management recommendations. Drugs Ther Perspect. (2020) 36:215–7. doi: 10.1007/s40267-020-00717-x, PMID: 32218651 PMC7095077

[17] McIntosh K Hirsch MS Bloom A. Coronavirus disease 2019 (COVID-19). Uptodate Hirsch MS Bloom. (2020) 5:873.

[18] Pradhan D Biswasroy P Kumar Naik P Ghosh G Rath G. A review of current interventions for COVID-19 prevention. Arch Med Res. (2020) 51:363–74. doi: 10.1016/j.arcmed.2020.04.020, PMID: 32409144 PMC7190516

[19] Peng D Wang Z Xu Y. Challenges and opportunities in mental health services during the COVID-19 pandemic. Gen Psychiatry. (2020) 33. doi: 10.1136/gpsych-2020-100275 PMC7462145 32914056

[20] EFMOH. National comprehensive covid19 management handbook. Ethiopian Federal Ministry of Health (2020).

[21] Teferi SC . Knowledge, attitude, and practice during the COVID-19 pandemic in Ethiopia: a review. International Journal of Clinical and Experimental Medical Sciences. (2020) 6:104. doi: 10.11648/j.ijcems.20200605.14

[22] Handebo S Adugna A Kassie A Shitu K. Determinants of COVID-19-related knowledge and preventive behaviours among students in reopened secondary schools: cross-sectional study. BMJ Open. (2021) 11:e050189. doi: 10.1136/bmjopen-2021-050189, PMID: 33895723 PMC8076628

[23] Larebo YM Abame DE. Knowledge, attitudes, and practices of face mask utilization and associated factors in COVID-19 pandemic among Wachemo university students, southern Ethiopia: a cross-sectional study. PLoS One. (2021) 16:e0257609. doi: 10.1371/journal.pone.0257609, PMID: 34543358 PMC8451998

[24] Angelo AT Alemayehu DS Dacho AM. Knowledge, attitudes, and practices toward COVID-19 and associated factors among university students in Mizan Tepi university. Infect Drug Resist. (2020) 14:349–60. doi: 10.2147/IDR.S299576 PMC7866950 33564243

[25] Berihun G Walle Z Teshome D Berhanu L Abebe M Ademas A . Knowledge, attitude, and preventive practices towards COVID-19 among students of Ethiopian higher education institutions. J Multidiscip Healthc. (2021) 14:2123–36. doi: 10.2147/JMDH.S322495, PMID: 34408427 PMC8364346

[26] Feleke A Adane M Embrandiri A Berihun G Walle Z Keleb A . Knowledge, attitudes, and misconceptions about COVID-19 prevention practices among high and preparatory school students in Dessie City, Ethiopia. J Multidiscip Healthc. (2022) 15:1035–55. doi: 10.2147/JMDH.S325636, PMID: 35586079 PMC9109976

[27] Tadesse AW Abebe NM Tadesse SE Wube MC Abate AA. Preventive practice and associated factors towards COVID-19 among college students in Amhara region, Ethiopia: a cross-sectional study. Ethiop J Health Sci. (2021) 31:3–14. doi: 10.4314/ejhs.v31i1.2, PMID: 34158747 PMC8188108

[28] Yesuf M Abdu M. Knowledge, attitude, prevention practice, and associated factors toward COVID-19 among preparatory school students in Southwest Ethiopia, 2021. PLoS One. (2022) 17:e0262907. doi: 10.1371/journal.pone.0262907, PMID: 35073358 PMC8786179

[29] Aklil MB Temesgan WZ. Knowledge and attitude towards COVID-19 vaccination and associated factors among college students in Northwest Ethiopia, 2021. Health Serv Res Manag Epidemiol. (2022) 9:23333928221098903. 35528024 10.1177/23333928221098903 PMC9073108

[30] Getawa S Aynalem M Bayleyegn B Adane T. Knowledge, attitude and practice towards COVID-19 among secondary school students in Gondar town, Northwest Ethiopia. PLoS One. (2022) 17:e0268084. doi: 10.1371/journal.pone.0268084, PMID: 35604938 PMC9126366

[31] Aynalem YA Akalu TY Gebresellassie Gebregiorgis B Sharew NT Assefa HK Shiferaw WS. Assessment of undergraduate student knowledge, attitude, and practices towards COVID-19 in Debre Berhan University, Ethiopia. PLoS One. (2021) 16:e0250444. doi: 10.1371/journal.pone.0250444, PMID: 34003825 PMC8130923

[32] Peters JP Hooft L Grolman W Stegeman I. Reporting quality of systematic reviews and meta-analyses of otorhinolaryngologic articles based on the PRISMA statement. PLoS One. (2015) 10:e0136540. doi: 10.1371/journal.pone.0136540, PMID: 26317406 PMC4552785

[33] Wubante SM Tegegne MD Melaku MS Walle AD Demsash. Knowledge sharing practice and its associated factors among health professionals in Ethiopia: systematic review and meta-analysis. Inform Med Unlock. (2022) 31:100967. doi: 10.1016/j.imu.2022.100967

[34] Walle AD Shibabaw AA Tilahun N Atinafu WT Adem JB Demsash AW . Readiness to use electronic medical record systems and its associated factors among health care professionals in Ethiopia: a systematic review and meta-analysis. Inform Med Unlock. (2022) 36:101140. doi: 10.1016/j.imu.2022.101140

[35] Tegegne MD Yilma TM Melaku MS Wubante SM Demsash AW Walle AD. Health information seeking and its associated factors in Ethiopia: systematic review and meta-analysis. Inform Med Unlock. (2022) 31:100980. doi: 10.1016/j.imu.2022.100980

[36] Mose A Zewdie A Sahle T. Pregnant women’s knowledge, attitude, and practice towards COVID-19 infection prevention in Ethiopia: a systematic review and meta-analysis. PLoS One. (2022) 17:e0276692. doi: 10.1371/journal.pone.0276692, PMID: 36288349 PMC9605027

[37] Yazew BG Abate HK Mekonnen CK. Knowledge, attitude and practice towards COVID-19 in Ethiopia: a systematic review. Patient Prefer Adherence. (2020) 15:337–48. doi: 10.2147/PPA.S288186 PMC7894797 33623375

[38] Lake EA Demissie BW Gebeyehu NA Wassie AY Gelaw KA Azeze GA. Knowledge, attitude and practice towards COVID-19 among health professionals in Ethiopia: a systematic review and meta-analysis. PLoS One. (2021) 16:e0247204. doi: 10.1371/journal.pone.0247204, PMID: 33606744 PMC7894858

[39] Azene AG Workie MS Aragaw AM. Knowledge, attitude, and prevention practices toward coronavirus disease 2019 in Ethiopia: a systematic review and meta-analysis. Curr Ther Res. (2021) 94:100633. doi: 10.1016/j.curtheres.2021.100633, PMID: 33972804 PMC8099548

